# Unveiling Anticancer Potential of COX-2 and 5-LOX Inhibitors: Cytotoxicity, Radiosensitization Potential and Antimigratory Activity against Colorectal and Pancreatic Carcinoma

**DOI:** 10.3390/pharmaceutics16060826

**Published:** 2024-06-18

**Authors:** Jelena Bošković, Vladimir Dobričić, Otilija Keta, Lela Korićanac, Jelena Žakula, Jelena Dinić, Sofija Jovanović Stojanov, Aleksandar Pavić, Olivera Čudina

**Affiliations:** 1 Department of Pharmaceutical Chemistry, University of Belgrade–Faculty of Pharmacy, Vojvode Stepe 450, 11000 Belgrade, Serbia; 2 Laboratory for Molecular Biology and Endocrinology, Vinča Institute of Nuclear Sciences, National Institute of the Republic of Serbia, University of Belgrade, Mike Petrovica Alasa 12-14, Vinca, 11351 Belgrade, Serbia; 3 Department of Neurobiology, Institute for Biological Research “Sinisa Stankovic”, National Institute of the Republic of Serbia, University of Belgrade, Bulevar Despota Stefana 142, 11108 Belgrade, Serbia; 4 Laboratory for Microbial Molecular Genetics and Ecology, Institute of Molecular Genetics and Genetic Engineering, University of Belgrade, Vojvode Stepe 444a, 11000 Belgrade, Serbia

**Keywords:** COX-2, 5-LOX, synthesis, cytotoxicity, radiosensitization, antimigratory potential, toxicity

## Abstract

Apart from cytotoxicity, inhibitors of the COX-2 enzyme have demonstrated additional effects important for cancer treatment (such as radiosensitization of tumor cells and cell antimigratory effects); however, the relationship between the inhibition of other inflammation-related enzyme 5-LOX inhibitors and anticancer activity is still not well understood. In our study, the cytotoxicity of thirteen COX-2 and 5-LOX inhibitors previously presented by our group (**1**–**13**) was tested on three cancer cell lines (HCT 116, HT-29 and BxPC-3) and one healthy cell line (MRC-5). Compounds **3**, **5**, **6** and **7** showed moderate cytotoxicity, but good selectivity towards cancer cell lines. IC_50_ values were in the range of 22.99–51.66 µM (HCT 116 cell line), 8.63–41.20 µM (BxPC-3 cell line) and 24.78–81.60 µM (HT-29 cell line; compound **7** > 100 µM). In comparison to tested, commercially available COX-2 and 5-LOX inhibitors, both cytotoxicity and selectivity were increased. The addition of compounds **6** and **7** to irradiation treatment showed the most significant decrease in cell proliferation of the HT-29 cell line (*p* < 0.001). The antimigratory potential of the best dual COX-2 and 5-LOX inhibitors (compounds **1**, **2**, **3** and **5**) was tested by a wound-healing assay using the SW620 cell line. Compounds **1** and **3** were singled out as compounds with the most potent effect (relative wound closure was 3.20% (24 h), 5,08% (48 h) for compound **1** and 3.86% (24 h), 7.68% (48 h) for compound **3**). Considering all these results, compound **3** stood out as the compound with the most optimal biological activity, with the best dual COX-2 and 5-LOX inhibitory activity, good selectivity towards tested cancer cell lines, significant cell antimigratory potential and a lack of toxic effects at therapeutic doses.

## 1. Introduction

Prolonged chronic inflammation is often associated with a range of serious diseases. The excessive generation of arachidonic acid (AA) cascade mediators, particularly those originating from the cyclooxygenase (COX) and lipoxygenase (LOX) pathways, is a crucial factor in the development of various inflammatory conditions. Inhibiting one biosynthetic pathway could lead to fatal consequences by shifting metabolism to the other. Consequently, compounds inhibiting both COX-2 and 5-LOX are believed to effectively block the production of inflammatory mediators from the AA pathway, offering a rational strategy for developing novel anti-inflammatory agents with enhanced safety profiles [1].

There are indications that inflammation is a critical element of tumor progression, with many cancers originating from sites of chronic irritation, infection, and inflammation. These insights are driving the development of innovative anti-inflammatory therapeutic approaches to cancer [2]. The connection between chronic inflammation and cancer was demonstrated in the context of colon carcinogenesis among individuals with inflammatory bowel diseases like chronic ulcerative colitis and Crohn’s disease [3].

Some studies indicate that long-term administration of non-steroidal anti-inflammatory drugs—NSAIDs (particularly aspirin) exhibit clinically meaningful anticarcinogenic effects in the gastrointestinal tract. These drugs decrease the risk for the development of colorectal cancer by about half [4]. According to one study, non-aspirin NSAIDs reduced colorectal risk after 6 months of continuous usage, while long-term usage of aspirin reduced risk only at doses of 300 mg daily [5]. While the precise mechanism of these effects of NSAIDs is not completely understood, it is proposed that COX-2 and 5-LOX enzymes are both simultaneously expressed and the resulting production of eicosanoids play significant roles in the development of these kind of cancers [6]. Dual inhibitors of the AA arachidonic acid pathway, such as licofelone, flavocoxid, and psoralidin, have been reported to have a mitigating effect on cancer progression [7]. The combined administration of celecoxib and the 5-LOX inhibitor MK886 significantly hinders the growth of pancreatic tumor cells in vitro, with reduced undesirable side effects [8]. It was shown that compounds that act as COX-2 inhibitors have cytotoxic effects against the hepatocellular carcinoma cell line [9].

The combined treatment of radiotherapy and selective COX-2 inhibitors represents a potentially more effective cancer treatment strategy. It was shown that celecoxib enhances tumor cell response to radiotherapy. Inhibition of the COX-2 enzyme was effective in activating proapoptotic pathways in numerous types of tumor cells [10]. The impact of selected COX-2 inhibitors on cell proliferation, when combined with radiation, seems to be dependent on the sequence of administration— radiosensitization was observed when inhibitors were applied before the radiation, while their administration after the radiation resulted in radioprotection [11]. On the other hand, the application of 12-LOX inhibitors might result in the radiosensitization of tumor cells, but there are no data on the effectiveness of 5-LOX inhibitors. Previous research has demonstrated that the overexpression of COX-2, and subsequent production of PGE_2_, can enhance cell motility in colorectal cancer cell lines [12]. Various studies examined the antimigratory potential of COX-2 inhibitors [12,13]. For the natural dual COX-2 and 5-LOX inhibitor psoralidin, potent cytotoxicity against HT-29 and MCF-7 cancer cell lines, as well as attenuation of fibroblast migration caused by radiation therapy, was demonstrated. Therefore, it might be useful as a potential lead compound in the development of new radioprotective agents against radiation-induced normal tissue injury [14,15].

The aim of this study was to investigate the cytotoxicity of thirteen COX-2 and 5-LOX inhibitors previously developed by our group [16], as well as to investigate its effects in combination with radiation, antimigratory potential, and the toxicity of selected inhibitors.

## 2. Materials and Methods

### 2.1. Tested Compounds

**Figure 1:**
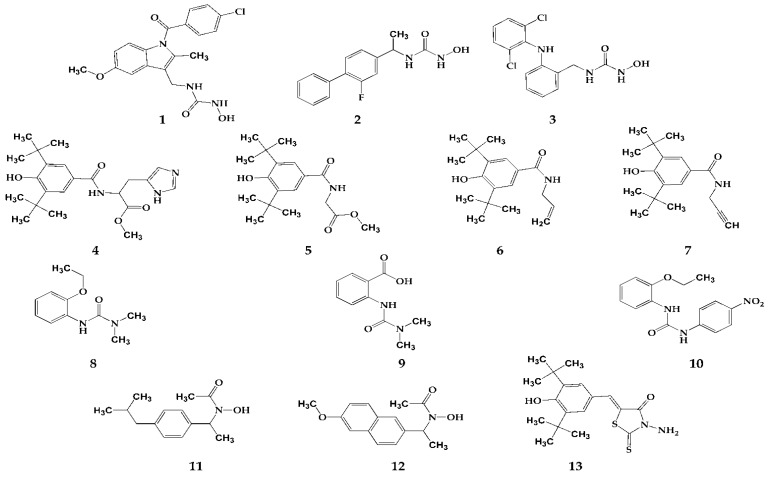
Structures of tested compounds **1**–**13**.

The synthesis of compounds **1**–**13** was presented in our previously published paper [16] and their chemical structures are presented in Figure 1. Compounds **1**–**3** belong to the group of N-hydroxyurea derivatives of well-known NSAIDs (indomethacin, flurbiprofen and diclofenac). Compounds **4**–**7** and compound **13** are derivatives of 3,5-di-tert-butyl-4-hydroxy benzene substituted in position C1. Compounds **8**–**10** are urea derivatives, while compounds **11** and **12** are “type B hydroxamic acids”.

The best dual COX-2 and 5-LOX inhibitors (compounds **1**, **2**, **3** and **5**) had IC_50_ values in the range of 5.26 µM to 18.28 µM (COX-2), and in the range of 1.62 µM to 9.30 µM (5-LOX). Other compounds are either COX-2 (compound **6** had the lowest IC50 value—6.89 µM) or 5-LOX inhibitors (compound **7** had the lowest IC50 value—12.55 µM) [16].

### 2.2. Evaluation of Cytotoxic Activity of Tested Compounds

#### 2.2.1. Cell Lines, Cell Culture and Treatments

Human colon cancer (HCT 116 and HT-29 cell lines), pancreatic cancer (BxPC-3 cell line), colorectal cancer (SW620 cancer cell line) as well as MRC-5 normal human fibroblasts and the corresponding cell culture media (McCoy’s 5A medium, RPMI-1640 medium and Eagle’s Minimum Essential Medium (EMEM)) were purchased from the American Type Culture Collection (ATCC, Rockville, MD, USA). Media for HCT 116, HT-29 and BxPC-3 cell lines was supplemented with 10% FBS (Gibco, Life Technologies, Carlsbad, CA, USA) and penicillin/streptomycin (Sigma-Aldrich Chemie GmbH, Steinheim, Germany) and maintained at 37 °C in a humidified atmosphere containing 5% CO_2_ (ESCO, Lifesciences Group, Singapore, Singapore). SW620 cells were cultured in Dulbecco’s Modified Eagle’s medium (DMEM, Sigma-Aldrich Chemie GmbH, Steinheim, Germany) and supplemented in the same manner as the previously mentioned cell lines. Cells were grown in cell culture flasks T25 (Sarstedt AG & Co., Nümbrecht, Germany) at appropriate cell densities in the culture medium prior to exposure to treatments. Stock solutions of each compound, prepared in DMSO, were diluted in the culture medium to obtain the appropriate final concentrations. The percentage of DMSO (Sigma-Aldrich Chemie GmbH, Steinheim, Germany) in the treated samples did not exceed 0.1% [17].

In the experiments involving irradiation, cells were irradiated at room temperature, in the vertical position with the cell monolayer facing ^60^Co γ-rays (CIRUS-Cis Biointernational). The irradiation of samples was performed in Vinča Institute of Nuclear Sciences, Belgrade, Serbia. In all experiments, a clinically relevant radiation dose of 2 Gy with the dose rate of ~1 Gy/min was applied.

#### 2.2.2. 2,5-Diphenyl-2H-tetrazolium Bromide (MTT) Assay

The viability of the control and cells treated with the tested compounds was determined by MTT assay [18]. Cells were seeded in 96-well plates at a density of 2000 cells/well, allowed to attach overnight, and then treated with rising concentrations of testing compounds. The ability of viable cells to reduce 3-(4,5-dimethylthiazol-2-yl)-2,5-diphenyltetrazolium bromide into blue-purple formazan crystals, was measured 72 h after the treatments. Namely, l mg/mL of MTT reagent (Sigma-Aldrich Chemie GmbH, Steinheim, Germany) was added to each well and incubated for an additional 4 h. Finally, upon removal of the medium containing MTT, 200 µL of DMSO was added to each well. The absorbance was measured using a microplate reader (Wallac VICTOR2 1420 Multilabel counter, PerkinElmer, Turku, Finland), at a test wavelength of 550 nm and a reference wavelength of 690 nm. The cell growth inhibition for each compound was expressed as an IC_50_ value, defined as the concentration that caused a 50% loss of cell growth.

#### 2.2.3. Sulforhodamine B (SRB) Assay

For the evaluation of cell viability after treatments with radiation as well as after combined treatments, a sulforhodamine B (SRB) protein binding assay was used [19]. This assay is based on the staining of cellular proteins. Maintaining conditions were identical to that of MTT assay. The cells were pretreated with IC_50_ concentrations of tested compounds for 24 h before irradiations. Following the 72 h post-irradiation incubation time under standard conditions (5% CO_2_, 37 °C) cells were fixed by protein precipitation with 10% trichloroacetic acid (TCA) at 4 °C for 1 h. Plates were rinsed with distillated water and stained with 0.4% SRB (Sigma-Aldrich Chemie GmbH, Steinheim, Germany) in 1% acetic acid, for 15 min at room temperature. Excess stain was removed by washing the wells in 1% acetic acid. After being rinsed, microwell plates were air dried until no staining moisture was visible. Bound dye was dissolved in 10 mM unbuffered Tris base (pH 10.5) for 5 min on a gyratory shaker. The absorbance of each well was measured using a microplate reader (Wallac VICTOR2 1420 Multilabel counter, PerkinElmer, Turku, Finland) at a test wavelength of 550 nm.

### 2.3. Evaluation of Antimigratory Potential of Selected Compounds

#### 2.3.1. Wound-Healing Assay

The migratory potential of SW620 cells (human colon carcinoma cell line) was evaluated by a wound-healing assay. Cells were seeded in density of 300,000 cells per well in 24-well tissue culture microplates and grown for 24 h in DMEM medium supplemented with 10% FBS, penicillin/streptomycin and l-glutamine at 37 °C in a humidified atmosphere containing 5% CO_2_. Upon reaching confluence, a uniform wound was scratched into a monolayer of each well with a sterile plastic 200 μL micropipette tip. After wounding, the medium was replaced and cells were further treated with 25 μM of compounds **1**, **2**, **3**, and **5** for 24 h and 48 h. Wound closure was monitored by imaging cells at 4× magnification using the ImageXpress^®^ Pico Automated Cell Imaging System (Molecular Devices^®^, San Jose, CA, USA) immediately (T0), and at 24 h (T24) and 48 h (T48) after wounding. The captured images were analyzed by ImageJ software v. 1.52p (U.S. National Institutes of Health, Bethesda, MD, USA) to measure the degree of closure of the wounded area. Independent experiments were performed three times.

#### 2.3.2. Calcein-AM Viability Assay

To evaluate cell viability, SW620 cells were prepared in the same manner as for the wound-healing assay. They were treated with 25 μM of compounds **1**, **2**, **3**, and **5** for 48 h and then co-stained using Calcein AM (Tocris Bioscience, Bristol, UK) and propidium iodide (Sigma-Aldrich Chemie Gmbh, Taufkirchen, Germany) for live/dead staining. Calcein AM staining is based on the hydrolysis of Calcein AM (Calcein acetoxy methyl ester), which is a non-fluorescent compound, to the bright green, fluorescent calcein hydrolyzed by cellular esterases. In this way, metabolically active cells in the sample can be detected. Unviable cells do not have cytoplasmic esterases; therefore, there is no appearance of green fluorescence. Propidium iodide (PI) is a dye used to stain dead cells with compromised membranes. The comparative use of Calcein AM and PI determines the percentage of viability of cells based on the number of metabolically active (green fluorescence) and dead (red fluorescence) cells. SW620 cells were incubated for 15 min at 37 °C in medium containing 4 μM Calcein AM and 5 μM propidium iodide. After washing in 1× PBS, the cells were imaged at 10× magnification using the ImageXpress^®^ Pico Automated Cell Imaging System (Molecular Devices^®^, San Jose, CA, USA). Quantification of live and dead cells was performed using CellReporterXpress^®^ software v. 2.8.2.669 (Molecular Devices^®^, San Jose, CA, USA).

### 2.4. Statistical Analysis

Results obtained in the MTT and SRB assays were presented as the mean ± SEM (standard error of the mean). The significance of differences among the experimental groups was assessed by the independent Student’s *t*-test, with the level of significance set at *p* < 0.05. Statistical analysis of the results obtained in the wound-healing and Calcein AM viability assays was performed by GraphPad Prism software v. 8.0.2 (San Diego, CA, USA). The results were analyzed by one-way analysis of variance (ANOVA) Dunnett’s multiple comparisons test, and the accepted level of significance was *p* < 0.05.

### 2.5. In Vivo Toxicity Assessment

All experiments involving zebrafish (*Danio rerio*) embryos were performed in compliance with the European directive 2010/63/EU and the ethical guidelines for the care and use of laboratory animals of the Institute of Molecular Genetics and Genetic Engineering, University of Belgrade. Wild-type (AB) zebrafish embryos, kindly provided by Dr. Ana Cvejić (Wellcome Trust Sanger Institute, Cambridge, UK), were raised to adult stage in a temperature- and light-controlled zebrafish facility at 28 °C and a standard 14:10 h light–dark photoperiod. Fish were fed with commercial dry food (SDS200 and SDS300 granular food; Special Diet Services, Essex; UK and TetraMinTM flakes; Tetra Melle, Germany) twice a day, and with *Artemia nauplii* daily.

The toxicity of compound **3**, diclofenac and zileuton was evaluated in accordance with the general rules of the OECD Guidelines for the Testing of Chemicals [20]. Embryos produced by pairwise mating were washed to remove debris, distributed at 10 per well into 24-well plates containing 1 mL of E3 medium (5 mM NaCl, 0.17 mM KCl, 0.33 mM CaCl_2_ and 0.33 mM MgSO_4_ in distilled water) and maintained at 28 °C. For assessing acute (lethal) and developmental (teratogenic) toxicity, the embryos were treated with the five different concentrations of the tested molecules (3.13, 6.25, 12.5, 25 and 50 µM) at 6 h post fertilization (hpf), an early embryonal stage ensuring high sensitivity to the applied molecules. Treated embryos were inspected every day under a stereomicroscope (Carl Zeiss™ Stemi 508 doc Stereomicro-scope, Jena, Germany) for the appearance of apical endpoints (Table S1) until 120 hpf, including survival, an appearance of developmental malformations and signs of cardiotoxicity and hepatotoxicity. Dead embryos were recorded and discarded every 24 h. DMSO (0.25%) and an E3 medium were used as the negative controls. Experiments were performed in triplicate using 30 embryos for each concentration. At 120 hpf, embryos were anesthetized by the addition of 0.1% (*w*/*v*) tricaine solution (Sigma-Aldrich, St. Louis, MO, USA), photographed, and killed by freezing at −20 °C for ≥24 h.

## 3. Results and Discussion

### 3.1. Evaluation of Cytotoxic Activities of Tested Compounds

Because the targeting of COX-2 and 5-LOX enzymes has been recognized as an important strategy in the therapy of colorectal and pancreatic carcinomas [8,21], cytotoxic activity was tested on colon (HCT 116 and HT-29) and pancreatic (BxPC-3) cancer cell lines, as well as on one healthy cell line (MRC-5). The cytotoxic activities of the tested compounds were expressed as IC_50_ values and are presented in Table 1. For comparison, three commercially available COX inhibitors (diclofenac, indomethacin and flurbiprofen) and one 5-LOX inhibitor (zileuton) were used. These standards were used because three out of the four best dual COX-2 and 5-LOX inhibitors (**1**, **2** and **3**) are their derivatives. According to the results, three compounds showed low cytotoxic activity against all tested cell lines. More specifically, compounds **8**, **9** and **12** demonstrated IC_50_ values higher than 100 µM. Other compounds showed fairly good cytotoxic effects, showing some variations between cell lines. Amongst these compounds, **1**, **4**, and **10** demonstrated a fairly strong cytotoxic effect both in the cancerous and healthy cell lines (Table 1). Compounds **1**, **2** and **3**, as derivatives of COX inhibitors indomethacin, flurbiprofen and diclofenac, respectively, were synthesized by the addition of 5-LOX pharmacophore into the structure of corresponding COX inhibitor. Data obtained in this study showed that dual COX-2 and 5-LOX inhibitors showed higher cytotoxicity against various cancer cell lines than corresponding clinical drugs, highlighting that chimeric molecules encompassing a COX-2 and a 5-LOX pharmacophore may be a promising strategy to increase anticancer activity and selectivity.

### 3.2. Evaluation of Radiosensitization Potential

**Figure 2:**
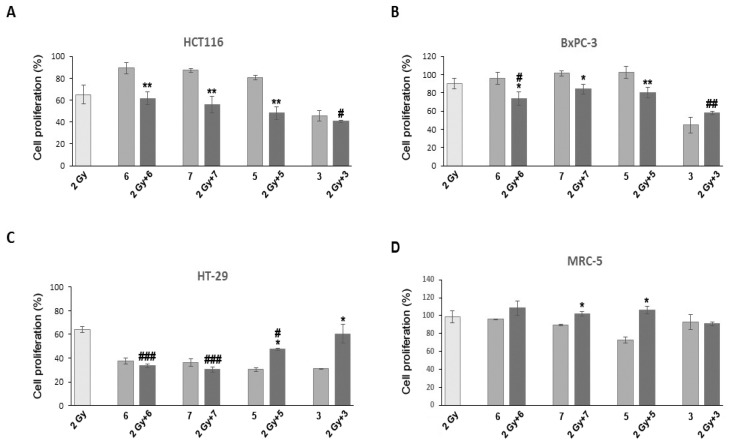
Effects of compounds **6**, **7**, **5**, **3** and γ-radiation (radiation dose of 2 Gy) on proliferation of HCT 116 (**A**), BxPC-3 (**B**), HT-29 (**C**), and MRC-5 (**D**) cells 72 h after the treatments. Bars are expressed as the mean of three independent replicate experiments ± SEM. Statistical significance was expressed as: ^*,#^ *p* < 0.05; **^,##^ *p* < 0.01; ***^,###^ *p* < 0.001. (*—Statistical significance compared to inhibitor; ^#^—statistical significance compared to radiation).

As COX-2 inhibition has been shown to be a potential strategy for increasing cancer cell response to radiation [10,11], the testing of combined treatments of selected compounds and radiation was performed using an SRB assay. It was shown that the SRB assay is effective for the in vitro testing of cancer cell sensitivity to radiation and for the study of interactions between radiotherapy and chemotherapy, with a sensitivity comparable to that of the standard clonogenic assay [22,23,24]. The selection of the best candidates for experiments employing radiation was based on their COX-2 and 5-LOX inhibitory effects, as well as on selectivity towards cancer cells. Compounds with strong cytotoxicity were excluded as the combined treatment would probably result in cellular “overkill”. Four compounds (**3**, **5**, **6** and **7**) met these criteria and were therefore chosen for further experiments. These compounds proved to be dual COX-2 and 5-LOX inhibitors, except **7**, which is a 5-LOX inhibitor. As can be seen from the results presented in Figure 2, radiation affected only HCT 116 and HT-29 cells to a significant extent with ~40% reduction in cell proliferation compared to the control, while MRC-5 and BxPC-3 cells did not respond significantly to treatments with radiation. Such a low rate of radiation-induced cell inactivation in these cell lines could be due to their inherent radiation resistance or to the effects of radiation in these cell lines tending to manifest at later time points [25,26].

All tested inhibitors had the most profound effect on HT-29 cells. However, other cell lines remained unaffected by most inhibitors (Figure 2A–D). These results were somewhat different from the results of the MTT assay, where four selected compounds at applied concentrations provoked a more than 50% decrease in cell proliferation. The observed differences between results obtained with SRB and MTT assays could be explained by differences in the assays applied [23]. Studies undertaken by several groups showed that results from SRB assay correlated well with those of MTT assay, although the IC_50_ values of compounds tested using SRB method were slightly higher [24,27,28,29]. The possible reason for these differences could be attributed to different endpoints that are measured by these assays. Namely, the MTT assay requires cellular metabolic activity to convert the colorless tetrazolium to the purple-colored formazan dye and, therefore, it detects only viable cells. By contrast, the SRB method is used for cell density determination and is based on the measurement of cellular protein content; thus, it does not distinguish between viable and dead cells. This difference, however, does not compromise the ability of SRB assay to detect the cytotoxic effect of a drug and/or radiation [24].

Although the combined treatment of compound **6** and radiation led to a decrease in cell proliferation in all cancer cell lines, a statistically significant change with respect to radiation was detected only in BxPC-3 and HT-29 cells (*p* ˂ 0.05 and *p* ˂ 0.001, respectively). Compared with radiation alone, compounds **5** and **7** only significantly inactivated HT-29 cells (*p* ˂ 0.05 and *p* ˂ 0.001, respectively) (Figure 2C). The rate of proliferation in compound **3** pretreated and irradiated HCT 116 cells was lower in comparison to cells that were only irradiated (*p* ˂ 0.05). This compound was also effective in the BxPC-3 cell line, with a significant change in cell inactivation when those two agents were applied in conjunction (*p* ˂ 0.01). Considering the intrinsic sensitivity of the cells to radiation and chemotherapeutics, the ability of the inhibitors used in this study to increase sensitivity to radiation might be cell-specific as it was shown that some of the cells (i.e., HT-29) were more sensitive than others. However, as shown in Figure 2B,C, compounds **3** and **5** were shown to have antagonistic effects with radiation. This could be explained by the different effects of these two agents on the cell cycle [30].

Studies in the literature about the effects of commercially available NSAIDs in combination with radiation shows that their radiosensitizing potential seems to be cell-type specific. For example, diclofenac demonstrated radiosensitizing activity against human colorectal cells (LS174T, LoVo), but not in lung (A549), breast (MDA-MB-231) and pancreatic (COLO357) carcinoma cells. Celecoxib enhances the radiosensitivity of HCT116 (human CRC cell line), while etodolac enhances the radiosensitivity of the HT29 cell line [31,32,33,34].

### 3.3. Evaluation of Antimigratory Potential of Selected Dual COX-2 and 5-LOX Inhibitors

Due to the existence of a relation between COX-2 inhibition and antimigratory activity, the best dual COX-2 and 5-LOX inhibitors (compounds **1**, **2**, **3** and **5**) were evaluated for their antimigratory potential by a wound-healing assay using the SW620 cell line. The cells were treated with 25 μM of tested compounds immediately after wounding, and wound closure was monitored immediately (T0), 24 h (T24), and 48 h after wounding (T48). Cell line SW620 has a strong metastatic and invasive potential, and therefore was chosen for the evaluation of the antimigratory potential of the tested compounds. In addition, this cell line was used in various studies that examined growth inhibitory effect of COX-2 inhibitors [35,36,37].

The obtained results are presented in Table 2, showing wound closure at T24 and T48 relative to T0, for each experimental condition.

**Figure 3:**
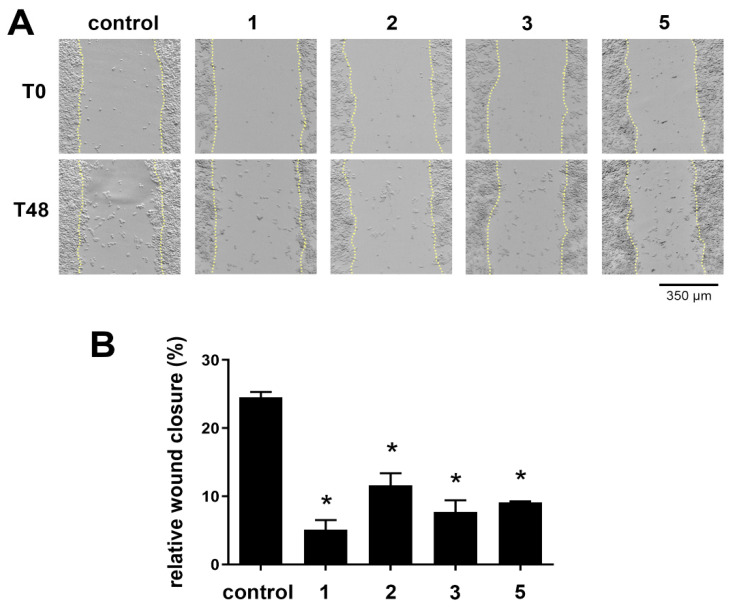
Migratory potential of SW620 cells assessed in the wound-healing assay: (**A**) representative images of each experimental condition taken at T0 and T48; and (**B**) the histogram is showing relative wound closure after 48 h treatment. Statistical significance compared to control is expressed as: * *p* < 0.05.

Based on the obtained results (Table 2), it can be concluded that the treatment with the tested compounds led to a decrease in cell migration after injury compared to the control (without treatment). The obtained results are also shown graphically, where statistically significant differences in the results are emphasized (Figure 3).

Statistically significant differences in cell migration after treatment with selected compounds compared to the control (without treatment) were observed for all tested compounds after 48 h. Three independent experiments were performed (n = 3) and the data are presented as mean ± SEM. The statistically significant difference between treatments and the untreated control is indicated by * *p* < 0.05 (Figure 3B).

Compared to the COX-2 inhibitor indomethacin, which significantly decreased the migratory ability of SW620 cells at a concentration of 400 μM after 36 h [38], compound **1** (indomethacin derivative) was more potent in its antimigratory property. In our study, the tested compound was applied at a lower concentration (25 µM) for 24 h, indicating that a shorter duration and a lower concentration are required to observe significant effects on cell migration. The higher potency of compound **1** suggests that this compound may offer advantages over indomethacin in terms of efficacy and efficiency. The COX-2 inhibitor flurbiprofen showed significant antimigratory effects on the SW620 cell line at a concentration of 10 nM after 24 h of treatment [39]. Compound **2,** which is a flurbiprofen derivative, showed activity in the micromolar range over the same time period. Compared to flurbiprofen, compound **2** showed weaker antimigratory activity. Furthermore, selective COX-2 inhibitor celecoxib at a final concentration of 30 μM applied to SW620 cells for 48 h showed comparable efficacy to compounds **1**, **2**, **3** and **5** in terms of antimigratory properties [40].

Based on these results, it can be concluded that all tested compounds exhibited an antimigratory effect, while the compounds **1** and **3** (N-hydroxy urea derivatives of indomethacin and diclofenac) stood out with their antimigratory potential.

To ensure that the observed antimigratory effect of the compounds was not the result of cell death, the Calcein AM viability assay based on live/dead cell staining was used to evaluate cell viability. The results are presented in Figure S1. Based on the obtained results, it can be concluded that after the applied treatment, there was no cell death caused by the treatment. The percentage of viable cells after all four treatments was approximately 100%, as with the untreated control. In addition, IC_50_ values of compounds **1**, **2**, **3**, and **5** following 24 h treatment are presented in Table S2.

Based on the obtained results of both tests, it can be concluded that the observed antimigratory effect was not a consequence of the cytotoxicity of the compounds applied at a concentration of 25 µM.

### 3.4. In Vivo Toxicity Assessment

To assess whether compound **3**, as the best drug candidate, can be used in a human population, its toxicity profile was investigated in vivo in the zebrafish (*Danio rerio*) model. Today, the zebrafish represents a universal biotechnological platform for the preclinical safety and efficacy assessment of new bioactive compounds as it has high genetic, molecular, physiological, and immunological similarity to mammals, including humans, and a high correlation in drug responses [41,42]. The use of this model system is also recommended by various regulatory bodies for early preclinical testing (i.e., FDA and NIH), simplifying the path to clinical trials and reducing the risk of failures in later stages of testing [43].

**Figure 4:**
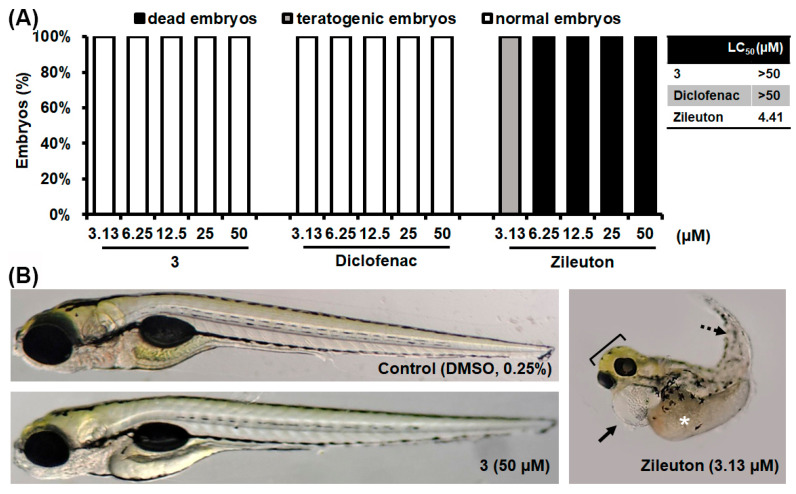
Toxicity of compound 3, diclofenac and zileuton assessed in vivo in the zebrafish model. (**A**) The dose-dependent effect on the embryos survival and teratogenicity. (**B**) Morphology of embryos exposed to 50 µM of compound 3 compared to the embryos treated with 3.13 µM zileuton and 0.25% DMSO (control). While compound 3 and diclofenac exhibited no adverse side effects, zileu-ton was very toxic causing life-treating pericardial edema (arrow), liver toxicity (asterisk), scoliosis (dashed arrow), and head malformations (bracket).

Here, wild-type (AB) zebrafish embryos were exposed to various doses of compound **3** for five days. Eighteen toxicity endpoints were examined (Table S1), covering all the most important signs of adverse side effects, among which liver toxicity (assessed by liver darkening and yolk absorption) and cardiotoxicity (manifested as enlargement of pericardial sac and disturbed heartbeat rate) are the most common side effects of new drugs in the preclinical development phase. The toxicity profile of this compound was evaluated in comparison to that of diclofenac and zileuton, clinically approved COX and 5-LOX inhibitors, respectively. As shown in Figure 4A, compound **3** did not elicit a toxic response in embryos exposed to any dose, similar to diclofenac. Namely, none of the exposed embryos showed signs of cardiovascular toxicity, hepatotoxicity, or skeletal malformations, in contrast to zileuton, which, at effective doses, caused numerous adverse side effects, such as life-threatening hepatotoxicity (liver and yolk necrosis), cardiotoxicity (pericardial edema) and various skeletal malformations (curved body, scoliosis, malformed head, jaw, eyes), leading to lethal outcomes after 5 days of exposure (Figure 4B). These data indicate that compound **3** was non-toxic at its therapeutic doses, including doses at which an anti-cancer effect (without or with irradiation, Figure 2) and an antimigratory effect (Figure 3) were achieved.

## 4. Conclusions

Thirteen previously synthesized COX-2 and 5-LOX inhibitors (compounds **1**–**13**) were tested for cytotoxicity using an MTT test on one healthy and three cancer cell lines. Compounds **3**, **5**, **6** and **7** proved to be good cytotoxic agents with good selectivity towards cancer cell lines. An investigation of the combined treatment of these four compounds and radiation was performed using an SRB assay. Radiation affected only HCT116 and HT-29 cells to a significant extent (with ~40% reduction in cell proliferation compared to the control), while all tested compounds had the most significant effect on HT-29 cells. The statistically most significant decrease in cell proliferation with respect to radiation was detected in the case of the combined treatment with compounds **6** and **7** in the HT-29 cell line (*p* < 0.001). The most potent dual COX-2 and 5-LOX inhibitors (**1**, **2**, **3** and **5**) were chosen for an investigation of antimigratory potential using a wound-healing assay and the SW620 cell line. Compounds **1** and **3** were singled out as compounds with the most potent antimigratory effect with a relative wound closure of 3.20% (24 h) and 5.08% (48 h) for compound **1** and 3.86% (24 h) and 7.68% (48 h) for compound **3**. Considering all these results, compound **3** stood out as the compound with the most optimal biological activity. It showed the best dual COX-2 and 5-LOX inhibitory activity, good selectivity towards the tested cancer cell lines and significant cell antimigratory potential. Finally, compound **3** did not show toxic effects at its therapeutic doses, including doses at which an anti-cancer effect (without or with irradiation) and an antimigratory effect were achieved.

## Figures and Tables

**Table 1:** Cytotoxic activities of tested compounds against one healthy and three cancerous cell lines.

Tested Compounds	IC50 Values (µM)			
	HCT 116	BxPC-3	HT-29	MRC-5
**1**	4.97 ± 0.78 (1.95) a	12.78 ± 1.11 (0.76)	9.78 ± 0.68 (0.99)	9.68 ± 2.34 (1)
**2**	64.76 ± 2.76 (1.07)	48.70 ± 4.60 (1.42)	82.38 ± 4.25 (0.84)	69.12 ± 3.07 (1)
**3**	40.76 ± 1.95 (>2.45)	34.28 ± 1.79 (>2.91)	64.64 ± 3.41 (>1.55)	>100 (/)
**4**	29.51 ± 5.23 (1.20)	24.72 ± 2.01 (1.43)	42.28 ± 2.57 (0.84)	35.44 ± 4.14 (1)
**5**	51.66 ± 1.78 (>1.94)	41.20 ± 2.88 (>2.43)	81.60 ± 2.77 (>1.22)	>100 (/)
**6**	22.99 ± 3.27 (>4.35)	8.63 ± 0.77 (>11.59)	24.78 ± 5.40 (>4.04)	>100 (/)
**7**	30.23 ± 5.07 (>3.31)	29.20 ± 1.56 (>3.42)	>100 (/)	>100 (/)
**8**	>100 (/)	>100 (/)	>100 (/)	>100 (/)
**9**	>100 (/)	>100 (/)	>100 (/)	>100 (/)
**10**	9.30 ± 0.38 (2.01)	6.05 ± 1.68 (3.09)	7.08 ± 0.30 (2.64)	18.68 ± 0.98 (1)
**11**	82.05 ± 2.34 (0.70)	63.02 ± 3.80 (0.91)	84.14 ± 3.43 (0.68)	57.10 ± 3.80 (1)
**12**	>100 (/)	76.68 ± 5.54 (0.83)	>100 (/)	63.56 ± 9.04 (1)
**13**	2.21 ± 0.12 (25.84)	1.80 ± 0.10 (31.72)	12.40 ± 1.95 (4.60)	57.10 ± 3.80 (1)
**Diclofenac**	79.11 ± 3.68 (1.14)	57.19 ± 3.03 (1.58)	74.95 ± 3.98 (1.21)	90.62 ± 3.20 (1)
**Indomethacin**	60.44 ± 4.27 (1.32)	85.91 ± 2.58 (0.93)	89.48 ± 4.45 (0.89)	79.76 ± 5.90 (1)
**Flurbiprofen**	˃100 (/)	˃100 (/)	˃100 (/)	˃100 (/)
**Zileuton**	44.89 ± 3.48 (2.13)	74.92 ± 4.10 (1.28)	78.49 ± 2.28 (1.22)	95.74 ± 0.94 (1)

^a^ Selectivity indexes (IC50 normal cell line/IC50 cancer cell lines) are presented in brackets.

**Table 2:** Relative wound closure after applied treatments at two time points (after 24 h and 48 h).

Tested Compounds	Relative Wound Closure (%)	
	24 h	48 h
** control **	17.87 ± 3.48	24.51 ± 1.39
** 1 **	3.20 ± 0.99	5.08 ± 2.46
** 2 **	7.47 ± 2.14	11.61 ± 3.06
** 3 **	3.86 ± 0.19	7.68 ± 2.99
** 5 **	7.12 ± 1.43	9.11 ± 0.27

## Data Availability

Data are contained within the article.

## Supplementary Materials

The following supporting information can be downloaded at: https://www.mdpi.com/article/10.3390/pharmaceutics16060826/s1, Figure S1: Viability of SW620 cells after 48 h treatment with compounds **1**, **2**, **3**, and **5**; Table S1: Lethal and teratogenic effects observed in zebrafish (*Danio rerio*) embryos at different hours post fertilization (hpf); Table S2: IC_50_ values of compounds **1**, **2**, **3**, and **5** following 24 h treatment in SW620 cells.
