# Supplementary material for: Unveiling Anticancer Potential of COX-2 and 5-LOX Inhibitors: Cytotoxicity, Radiosensitization Potential and Antimigratory Activity against Colorectal and Pancreatic Carcinoma
Source: Pharmaceutics. 2024 Jun 18;16(6):826. doi: 10.3390/pharmaceutics16060826 (PMC11207729; doi:10.3390/pharmaceutics16060826)
Supplement: Supplementary file 1 [file pharmaceutics-16-00826-s001.zip › pharmaceutics-3004580-supplementary.pdf]

Supplementary Information associated with the paper

Unveiling Anticancer Potential of COX-2 and 5-LOX Inhibitors: Cytotoxicity, Radiosensitization Potential and Antimigratory Activity Against Colorectal and Pancreatic Carcinoma

Jelena Bošković[1], Vladimir Dobričić[1*], Otilija Keta[2], Lela Korićanac[2], Jelena Žakula[2], Jelena Dinić[3], Sofija Jovanović Stojanov[3], Aleksandar Pavić[4], Olivera Čudina[1]

[1] Department of Pharmaceutical Chemistry, University of Belgrade – Faculty of Pharmacy, Vojvode Stepe 450, 1100 Belgrade, Serbia
[2] Vinča Institute of Nuclear Sciences, National Institute of the Republic of Serbia, University of Belgrade, Mike Petrovica Alasa 12-14, 11351 Vinca, Belgrade, Serbia
[3] Department of Neurobiology, Institute for Biological Research "Sinisa Stankovic", National Institute of the Republic of Serbia, University of Belgrade, Bulevar Despota Stefana 142, 11108 Belgrade, Serbia
[4] Laboratory for Microbial Molecular Genetics and Ecology, Institute of Molecular Genetics and Genetic Engineering, University of Belgrade, Vojvode Stepe 444a, 11000 Belgrade, Serbia
*Correspondence: vladimir@pharmacy.bg.ac.rs

Contents

Figure S1. Viability of SW620 cells after 48h treatment with compounds **1**, **2**, **3**, and **5**. 2

Table S1. Lethal and teratogenic effects observed in zebrafish (Danio rerio) embryos at different hours post fertilization (hpf). 3

Table S2. IC_{50} values of compounds **1**, **2**, **3**, and **5** following 24h treatment in SW620 cells. 3

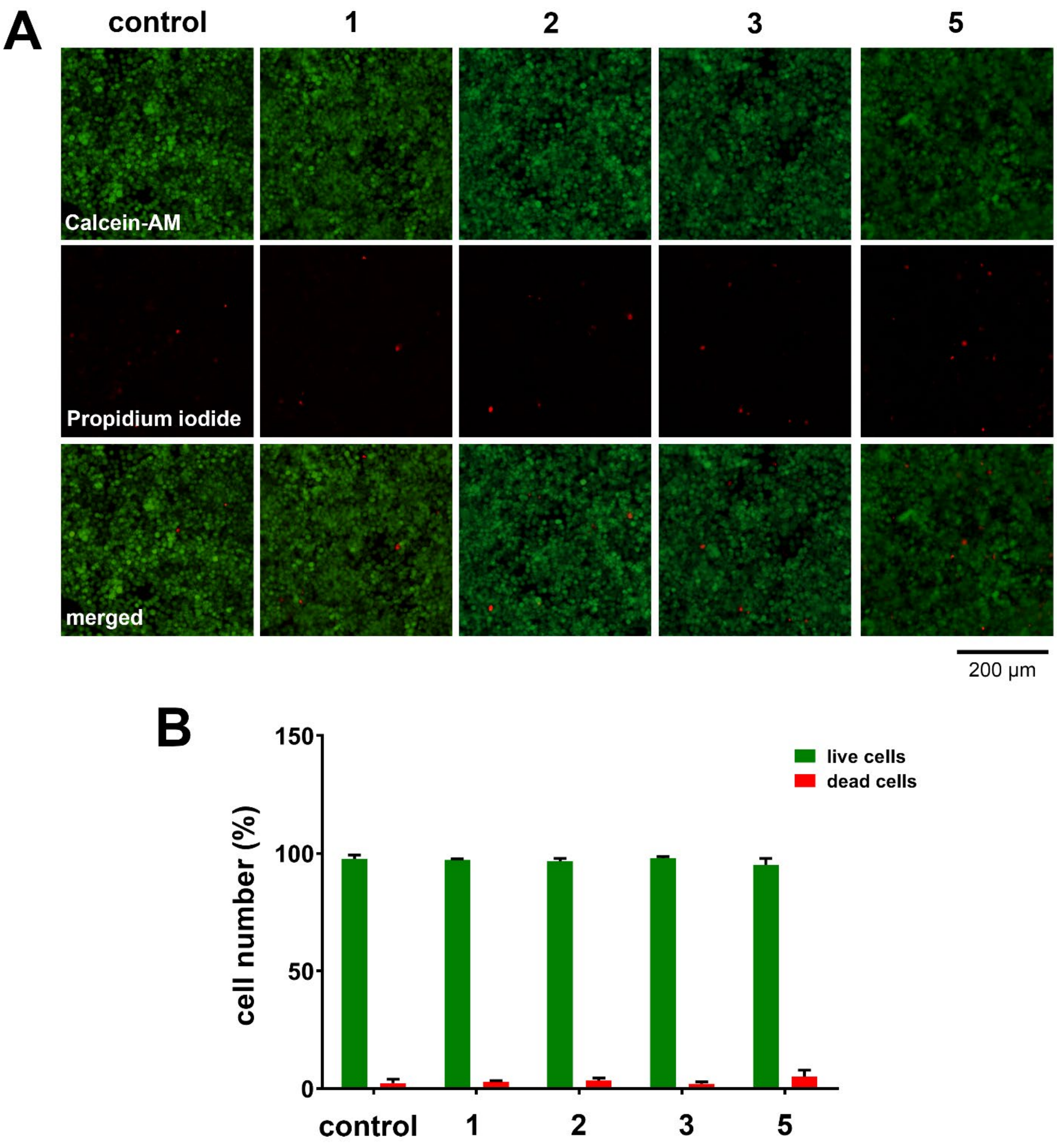

Figure S1. Viability of SW620 cells after 48h treatment with **1**, **2**, **3**, and **5**: (A) representative images; and (B) quantification of Calcein-AM-labeled (live) and propidium-iodide-labeled (dead) SW620 cells after 48 h treatment with 25 μM **1**, **2**, **3**, and **5**. The average number of live/dead cells per field was determined. Data are presented as mean ± SD (n = 3).

Table S1. Lethal and teratogenic effects observed in zebrafish (*Danio rerio*) embryos at different hours post fertilization (hpf).

Category	Toxicological parameters	Exposure time (hpf)				
		24	48	72	96	120
**Lethal effect**	Coagulated eggs[a]	●	●	●	●	●
	Lack of the heart beating	●	●	●	●	●
	Non-detachment of the tail	●	●	●	●	●
	Lack of somite formation	●	●	●	●	●
**Teratogenic effect**	Malformation of head	●	●	●	●	●
	Malformation of eyes[b]	●	●	●	●	●
	Malformation of sacculi/otoliths[c]	●	●	●	●	●
	Malformation of chorda[d]	●	●	●	●	●
	Malformation of tail[e]	●	●	●	●	●
	Scoliosis/lordosis	●	●	●	●	●
	Yolk edema[f]	●	●	●	●	●
	Growth retardation[g]		●	●	●	●
	Hatching[h]			●	●	●
	Swimmbladder development[i]					●
**Hepatotoxicity**	Yolk absorption[j]			●	●	●
	Liver darkening[k]			●	●	●
**Cardiotoxicity**	Pericardial edema[l]		●	●	●	●
	Heart beating rate (beat/min)[m]					●

[a] No clear organs structure is recognized
[b] Malformation of eyes was recorded for the retardation in eye development and abnormality in shape and size.
[c] Presence of none, one or more than two otoliths per sacculus, as well as reduction and enlargement of otic vesicles
[d] The abnormality in notochord shape
[e] Tail malformation was recorded when the tail was bent, twisted or shorter than to control embryos as assessed by optical comparison
[f] Enlargement of the yolk sac
[g] Growth retardation was recorded by comparing with the control embryos in a body length (after hatching)
[h] Embryos hatching in a period from 72 hpf to 120 hpf stage
[i] The presence, reduced size or absence of swimmbladder
[j] The resorption of yolk
[k] The change in liver color and dark color appearance
[l] An appearance of pericardial sac enlargment
[m] The number of beats within 30 sec

Table S2. IC_{50} values of compounds **1**, **2**, **3**, and **5** following 24h treatment in SW620 cells.

Compound	IC_{50} (μM)
**1**	215.1 ± 4.9
**2**	386.3 ± 11.8
**3**	168.7 ± 5.7
**5**	131.8 ± 3.5
